# Macular hole with inner limiting membrane peeling off spontaneously in Terson syndrome: A case report

**DOI:** 10.1097/MD.0000000000025960

**Published:** 2021-06-04

**Authors:** Hui Qi, Hongtao Yan, Yan Cheng, Ling Zuo

**Affiliations:** Eye Center of the second hospital, Jilin University, ChangChun, Jilin Province, China.

**Keywords:** inner limiting membrane, macular hole, pars plana vitrectomy, Terson syndrome, vitreous hemorrhage

## Abstract

**Introduction:**

Terson's syndrome with inner limiting membrane (ILM) peeled off spontaneously is rarely seen, and the mechanism of it is not clear. Here we report a case of Terson Syndrome with a rare finding: the ILM peeled off spontaneously associated with macular hole (MH).

**Patient concerns:**

A 36-year-old female patient was admitted to our hospital with decreased visual acuity in the right eye lasting for 1 month. She just had surgery for subarachnoid hemorrhage that occurred 1 month before due to the rupture of the intracranial aneurysm.

**Diagnosis:**

Terson syndrome was diagnosed according to her medical history and examination. A partial posterior vitreous detachment (PVD) and dense vitreous hemorrhage (VH) was confirmed in the right eye by performing ophthalmic B-scan ultrasonography examination. Head computed tomography showed the subarachnoid hemorrhage after aneurysmal rupture.

**Interventions:**

The patient underwent pars plana vitrectomy in her right eye to remove the VH. After removal of the VH, a full-thickness macular hole was noted with the ILM peeled off spontaneously. So we conducted gas tamponade, and face-down positioning after pas plana vitrectomy.

**Outcomes:**

At two weeks follow-up, her best corrected visual acuity was 0.15 in the right eye. Spectral domain optical coherence tomography showed that the MH was closed completely, while the thickness of the nasal retina of the foveal was thicker than that on the temporal side.

**Lessons:**

ILM peeled off spontaneously associated with MH is a rarely seen complication of Terson Syndrome. Due to the large-scale of the ILM peeling off, final visual acuity may be poor in patients, even though successful macular hole closure after the operation.

## 1 Introduction

Terson syndrome is a condition characterized by intraocular hemorrhage due to subarachnoid hemorrhage (SAH) in association with acutely elevated intracranial pressure,^[1]^ and vitreous hemorrhage (VH) is the major symptom.^[2]^ In most cases, the hemorrhage is simple and can be removed by timely pars plana vitrectomy (PPV) with immediate improvement of vision.^[3]^ However, there may be multiple complications, such as macular epiretinal membrane, retinal detachment, retinal folds, and macular holes (MHs). However, the inner limiting membrane (ILM) peeling off spontaneously during PPV for VH has never been reported.

We describe the case of a patient with Terson syndrome accompanied by VH in the right eye and in whom an MH was found during PPV. We also found that the ILM within the macular region had already peeled off spontaneously during surgery. To the best of our knowledge, there are no reports on this association. Here, we report a case of Terson syndrome with the ILM peeling off spontaneously associated with MH.

## 2 Case presentation

**Figure 1:**
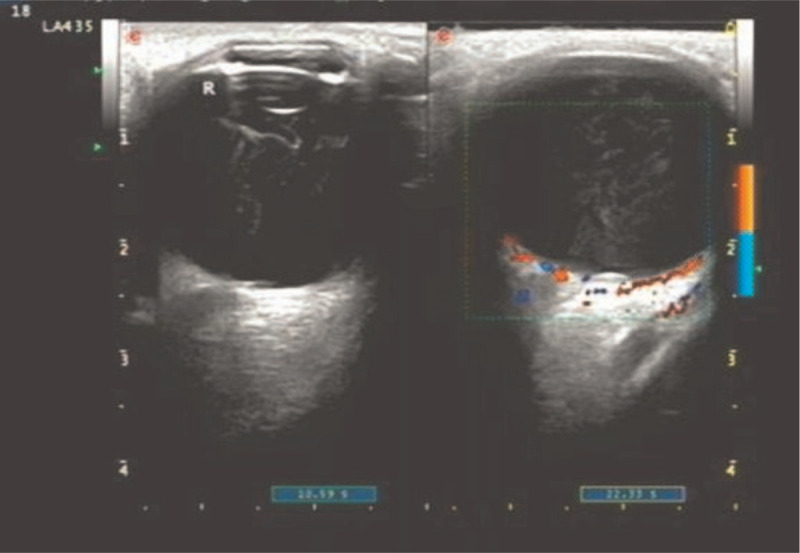
Ultrasonography image of the right eye, with a partial posterior vitreous detachment and dense vitreous hemorrhage.

**Figure 2:**
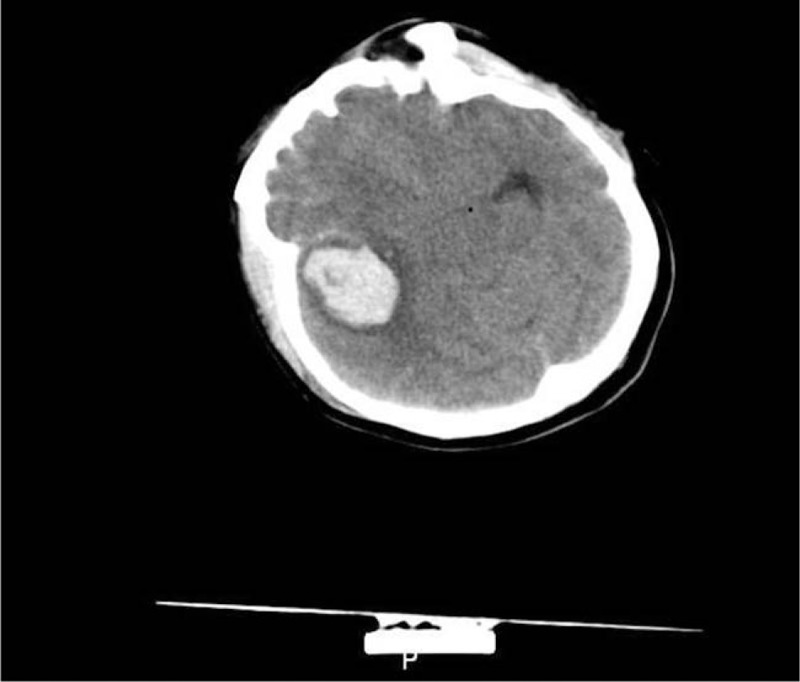
Head computed tomography showing the subarachnoid hemorrhage after aneurysmal rupture.

A 36-year-old female patient was admitted to our hospital with decreased visual acuity in the right eye that lasted for 1 month. She had just undergone surgery for SAH that occurred 1 month before due to rupture of an intracranial aneurysm. Before the SAH, she did not notice any changes in her vision in either eye. An eye examination was performed by an ophthalmologist; the best corrected visual acuity (BCVA) was hand motion in the right eye and 1.0 in the left eye (standard logarithmic visual acuity chart, Chinese edition). Intraocular pressure and anterior segment examinations were unremarkable. Fundus examination revealed a massive VH in the right eye. Partial posterior vitreous detachment (PVD) and dense VH were confirmed in the right eye by performing ophthalmic B-scan ultrasonography (Fig. 1). Head computed tomography revealed SAH after aneurysmal rupture (Fig. 2). Terson syndrome was diagnosed based on the patient's medical history and examination.

**Figure 3:**
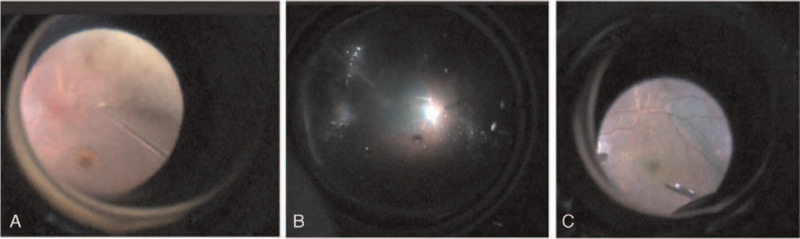
Images of the right eye after vitrectomy. A: After removal of the vitreous hemorrhage, a full-thickness macular hole was noted. B: 0.25% indocyanine green was used to stain the inner limiting membrane (ILM). C: The ILM had already peeled spontaneously, ranging from the upper to lower vascular archese. ILM = inner limiting membrane

The patient underwent PPV in the right eye to remove the VH. After removal of the VH, a full-thickness MH was noted, and the remainder of the retina appeared normal after PPV (Fig. 3A). In order to peel the ILM, fluid-air exchange was performed, and 0.25% indocyanine green (ICG) was injected slowly under air (Fig. 3B). To our surprise, the region ranging from the upper vascular arch to the lower vascular arch had not been stained, while the other part of the posterior pole of the fundus was stained well (Fig. 3C), which means that the ILM had peeled spontaneously. Therefore, we conducted gas tamponade and face-down positioning after PPV.

**Figure 4:**
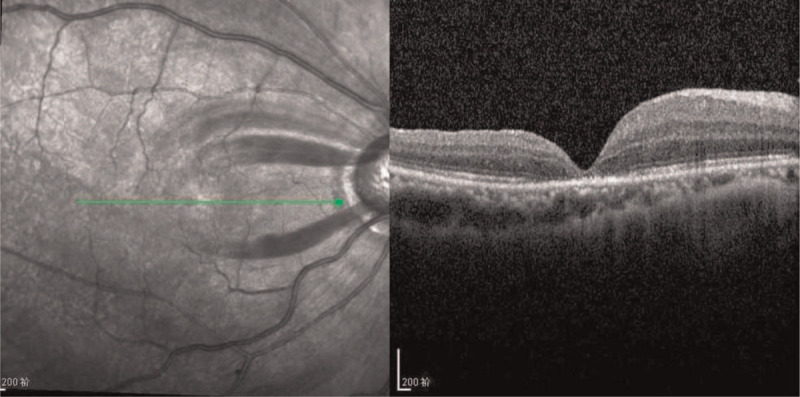
Optical coherence tomography of the right eye at two weeks after vitrectomy. The macular hole was completely closed. The retinal thickness in the nasal side of the foveal is 395 nm and the temporal side is 258 nm.

Both BCVA and spectral domain optical coherence tomography were recorded at the 2-week follow-up. Her BCVA was 0.15 in the right eye and 1.0 in the left eye, respectively. Spectral domain optical coherence tomography showed that the MH was closed completely, while the thickness of the nasal retina of the fovea was thicker than that on the temporal side (Fig. 4). The patient was satisfied with the improvement in postoperative vision.

## 3 Discussion

Terson syndrome is defined as VH secondary to SAH or intracranial hemorrhage.^[4]^ The clinical classification of intraocular hemorrhage according to the potential location of the hemorrhage has been described as either submembranous (sub-ILM), preretinal (between the ILM and posterior hyaloid), retinal, subhyaloid, or intravitreal.^[5]^ In our case, after removal of the dense VH, we could clearly see the dissection of the ILM and the retina, as confirmed by staining with ICG during PPV. Sub-ILM hemorrhage is often observed in patients with Terson syndrome. Munteanu et al. reported a case of bilateral Terson syndrome with a sub-ILM hemorrhage.^[6]^ Abed Alnabi et al. reported a case in which the appearance of perimacular folds associated with rapid accumulation of blood in the sub-ILM space was considered mainly due to the anterior-posterior traction of the macular ILM.^[7]^ However, there has never been a case reported where the ILM within the macular region had peeled off spontaneously. In our case, the ILM was completely peeled off, and the region ranged from the upper vascular arch to the lower vascular arch. To date, the mechanism of blood entrance is not clear. Several theories may explain the pathogenesis of Terson syndrome. First, the blood from the SAH extends directly into the vitreous space through the intervaginal space around the optic nerve by penetrating the lamina cribrosa of the sclera.^[8]^ The other possible mechanism is that SAH induces sudden intracranial hypertension, which results in stasis of the retinal veins and induces rupture of the retinal veins or peripapillary capillaries.^[9]^ Our hypothesis is that the amount of sub-ILM hemorrhage was large enough to cause VH in a short period of time. As a consequence, the ILM was abruptly torn off, and a large amount of blood spread into the vitreous cavity.

A full-thickness MH found during PPV was another rare complication in our case. Rubowitz and Desai described two patients with non-traumatic Terson syndrome who were found to have MHs during PPV for non-clearing VH. He believed that the formation of epiretinal membranes may be responsible for this common association.^[10]^ It is well-accepted that the mechanism of MH is tangential anterior-posterior traction of the vitreomacular or macular ILM.^[11]^ Moreover, a sudden bloody dissection of the ILM may produce tractional forces responsible for causing MH. In this case, the ILM was not peeled surgically. Therefore, we concluded that the pathogenic mechanisms of this unusual MH may be due to anterior-posterior traction on the fovea by ILM thickening or peeling.

Another sign that should not be ignored is postoperative OCT. OCT showed that the MH was closed, while the retinal thickness on the nasal side of the macular central fovea was much thicker than that on the temporal side. This may be because the traction on the nasal side is stronger than that on the temporal side, by which we infer that the hemorrhage under the ILM on the nasal side was greater than that on the temporal side. This sign seems to support the first theory that blood comes from the edge of the optic nerve.

Above all, we infer that the ILM peeled off spontaneously, and the MH in our case was due to macular ILM traction due to a sudden large amount of blood accumulating under the ILM in a short period of time. For this reason, we are ready to accept the first theory of the previously proposed mechanisms of Terson syndrome, which suggests that blood from an SAH extends directly into the vitreous space through the intervaginal space around the optic nerve by penetrating the lamina cribrosa of the sclera. This is difficult to explain by the second theory, which proposes that the blood originates from the ruptured retinal veins or the peripapillary capillaries, such as central retinal vein occlusion. Large and flame-shaped retinal hemorrhages rarely occur in central retinal vein occlusion patients.

## 4 Conclusions

In conclusion, we should consider that the spontaneous peeling of the ILM associated with MH is also a complication of Terson syndrome, although it rarely happens. A sudden bloody dissection of the ILM may produce tractional forces responsible for causing MH. Due to the large-scale peeling of the ILM, the final visual acuity may be poor in patients, even after successful MH closure.

## Acknowledgments

The authors thank Editage (www.editage.com) for their English language editing service.

## Author contributions

**Project administration:** Yan Cheng.

**Software:** Hongtao Yan.

**Writing – original draft:** Hui Qi.

**Writing – review & editing:** Ling Zuo.
